# Global Population Exposed to Extreme Events in the 150 Most Populated Cities of the World: Implications for Public Health

**DOI:** 10.3390/ijerph18031293

**Published:** 2021-02-01

**Authors:** Linze Li, Chengsheng Jiang, Raghu Murtugudde, Xin-Zhong Liang, Amir Sapkota

**Affiliations:** 1 Maryland Institute for Applied Environmental Health, University of Maryland School of Public Health, College Park, MD 20742, USA; linzeli.whu@gmail.com (L.L.); cjiang89@umd.edu (C.J.); 2 School of Remote Sensing and Information Engineering, Wuhan University, Wuhan 430079, China; 3 Earth System Science Interdisciplinary Center, University of Maryland, College Park, MD 20742, USA; mahatma@umd.edu (R.M.); xliang@umd.edu (X.-Z.L.)

**Keywords:** climate change, extreme heat event, extreme precipitation event, megacities, urban area, urban heat island effect, global population

## Abstract

Climate change driven increases in the frequency of extreme heat events (EHE) and extreme precipitation events (EPE) are contributing to both infectious and non-infectious disease burden, particularly in urban city centers. While the share of urban populations continues to grow, a comprehensive assessment of populations impacted by these threats is lacking. Using data from weather stations, climate models, and urban population growth during 1980–2017, here, we show that the concurrent rise in the frequency of EHE, EPE, and urban populations has resulted in over 500% increases in individuals exposed to EHE and EPE in the 150 most populated cities of the world. Since most of the population increases over the next several decades are projected to take place in city centers within low- and middle-income countries, skillful early warnings and community specific response strategies are urgently needed to minimize public health impacts and associated costs to the global economy.

## 1. Introduction

Ongoing climate variability and change are increasing the frequency, duration, and intensity of extreme weather events across the globe [1,2]. Recent estimates suggest that these threats will cause approximately 250,000 deaths per year between 2030 and 2050 with direct damage costing $2–4 billion per year [1,3]. Extreme heat events (EHE) and extreme precipitation events (EPE) are important attributes of climate variability and climate change that pose direct threats to public health across the globe. Previous studies have shown that exposure to extreme heat can increase the risk of mortality [4,5,6,7,8] and range of morbidity, including cardiopulmonary health outcomes [9,10,11,12,13]. Likewise, the increases in frequency of extreme precipitation events that are tied to warming climate can induce flooding, increase the risks of landslides, and degrade water quality [14,15]. Besides directly increasing the risk of death from drowning, EPE can also enhance the fecal-oral route of exposure to many pathogenic microorganisms and increase risk of food and waterborne illness [16,17,18,19,20]. In addition, EHE and EPE can also indirectly impact biodiversity, rainforest ecosystem deterioration, and food insecurity, which are all tied to human disease risks [1,2,21,22,23,24].

Increases in frequency of EHE and EPE may be higher in urban areas characterized by high population density and worsening air quality [25,26,27]. The increasing trend of EHE and EPE in these population centers is further exacerbated by the urban heat island effect that results from large areas of impervious land cover and low vegetation coverage [26,28,29,30,31,32,33,34]. This is particularly concerning to public health as the proportion of the global population residing in large urban centers continues to grow every year, with the most recent United Nation report suggesting that 68% of the world population will be living in such urban areas by 2050 [35]. As the world continues to urbanize, many megacities are facing challenges in meeting the needs of their growing urban populations, including housing, transportation, energy systems and other infrastructure, as well as employment and basic services such as education, health care and higher rates of urban poverty [36,37,38]. In addition, these urban centers are also notorious for disparities that exist with regards to income, food, and healthcare access [39,40,41,42,43]. The confluence of these factors make urban areas more vulnerable to threats posed by climate change related increases in extreme weather events [44]. Yet, there is a paucity of data regarding how frequency of EHE and EPE are being felt across the major population centers of the world. Currently, over one billion people reside in just 150 of the most populated cities. Most studies that have investigated the impacts of climate change on extreme events and resulting adverse human health impacts in urban population centers have originated primarily from developed countries with few exceptions [45,46,47]. This critical data gap limits the development of robust adaptation plans in cities within low- and middle-income countries where they are most urgently needed.

To address these issues, we characterized the frequency of EHE and EPE between 1980 and 2017 across the 150 most populated metropolitan locations of the world and estimated the trend in exposed populations using meteorological data from monitoring stations as well as climate models. We further analyzed this data to better characterize seasonal, regional, and temporal trends.

## 2. Materials and Methods

### 2.1. Population Data

We acquired information on the 150 most populated metropolitan locations of the world with populations over 3 million from the World Atlas [48] and downloaded their boundaries from Open Street Map [49] using python software (python 2.7.1). We obtained metropolitan human population data for these cities from the United Nations Human Population Division (UNPD) [50]. We then used a simple linear interpolation method to estimate yearly populations as described previously [51]. We divided the 150 global cities into four zones: North Temperate (latitude between 23.5° N and 66.5° N); North Tropic (0° N and 23.5° N); South Temperate (23.5° S and 66.5° S); South Tropic (0S and 23.5° S). We also grouped these global cities according to the World Health Organization (WHO) classification system into [52]: African Region; Region of the Americas; South East Asia Region; European Region; Eastern Mediterranean Region; and Western Pacific Region.

### 2.2. EHE and EPE Metrics

We obtained daily maximum temperature (TMAX) and precipitation data for 1980–2017 from the Global Historical Climatology Network (GHCN) through the National Climate Data Center (NCDC) data portal (https://www.ncdc.noaa.gov). If a metropolitan location had multiple meteorological stations, we used the one closest to the urban core. If no station data were available, we borrowed information from stations that were located within a 50 km radius of the location boundary or set it to “missing” if no stations were available within the 50 km radius. We used 20 years (01/1980–12/1999) of daily meteorological data (maximum temperature, precipitation) as the base period to calculate calendar day and location-specific 95th percentile thresholds to define EHE and EPE. For example, the baseline data for Beijing for January 16th consisted of all daily observations for Beijing from 1 January to 31 January from 1980 to 1999. If a metropolitan area had more than 30% of daily observations missing during the baseline period (1980–1999), we dropped them from the analysis. Based on the distribution of this data, we identified location and calendar day specific 95th percentile threshold values for TMAX and precipitation, referred to as EHE_95_, and EPE_95_ thresholds, respectively. Calendar day-specific TMAX and precipitation values for each metropolitan region were compared with their respective EHE_95_ and EPE_95_ thresholds and assigned a value of “1” if they exceeded the threshold, and “0” otherwise [18,53]. Days where TMAX exceeded EHE_95_ were identified as extreme heat events, and days where the daily total precipitation exceeded the EPE_95_ thresholds were identified as extreme precipitation events.

**Figure 1:**
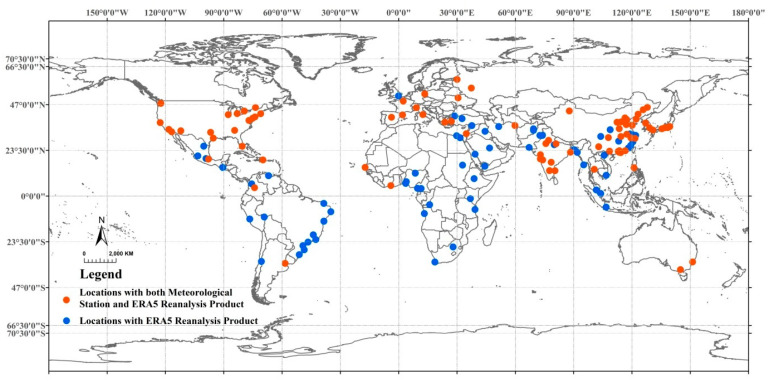
Global distribution of the 150 most populated metropolitan locations.

Since numerous cities did not have weather station data satisfying our inclusion criteria, we also performed additional analysis using ECMWF ERA5 reanalysis products with 0.25 × 0.25 degree spatial resolution [54]. We used the hourly ECMWF ERA5 products to calculate daily TMAX and total precipitation. Based on these data, we calculated EHE and EPE for all 150 cities using the aforementioned method (Figure 1, Supplementary Table S1).

To harmonize the definition of season between the Northern and Southern Hemisphere, we defined June, July and August as summer in the Northern Hemisphere and winter in the Southern Hemisphere. Likewise, December, January and February were classified as winter in the Northern hemisphere and summer in the Southern Hemisphere. Spring (March, April, and May) and fall (September, October, and November) definitions followed suit. We calculated seasonal and yearly sums of EHE and EPE for all metropolitan locations. We computed yearly exposed populations for each city based on the yearly population and frequency of EHE and EPE for the respective cities (Supplementary Equation S1). We also characterized EHE and EPE frequency distribution across seasons (Spring, Summer, Fall, Winter), by latitudes (North Temperate, North Tropic, South Temperate, South Tropic), and the WHO regions (Africa, Eastern Mediterranean, Europe, Region of the Americas, South East Asia, and Western Pacific Region). Finally, we applied ordinary least squares (OLS) regression to estimate seasonal increases in rates of EHE and EPE frequency across these regions and time periods.

## 3. Results

**Figure 2:**
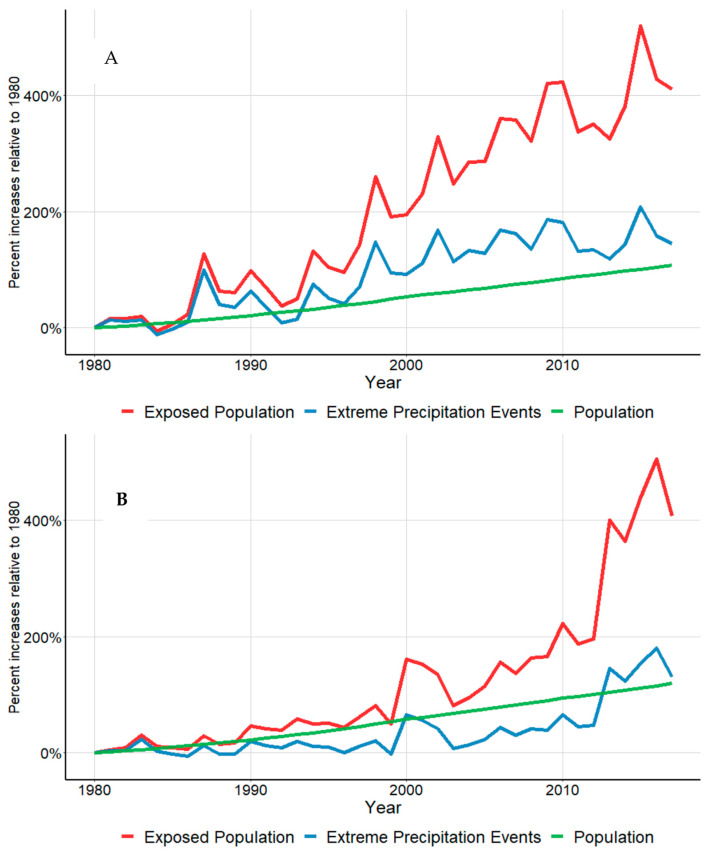
Changes in EHE (**A**) and EPE (**B**) exposed populations during the last four decades.

Of the 150 largest cities, 84 had valid weather data during the 1980–2017 period that satisfied our inclusion criteria. On average, these 84 cities were missing less than 8% of daily observations (Supplementary Table S1). For the analysis involving ECMWF ERA5 reanalysis products, we used all 150 metropolitan locations. As depicted in Figure 2, there was a clear increasing trend in frequencies of EHE and EPE across the major city centers of the world during the last four decades (1980–2017). These increases, combined with the steady growth in urban populations in these 150 locations translated into a 500% increase in the number of urban populations exposed to EHE and EPE during the four decades. When this analysis was repeated using ECMWF ERA5 reanalysis products, the percent increase in urban populations exposed to EHE was similar, however this was not the case for populations exposed to EPE (~150% increase, Supplementary Figure S1).

**Figure 3:**
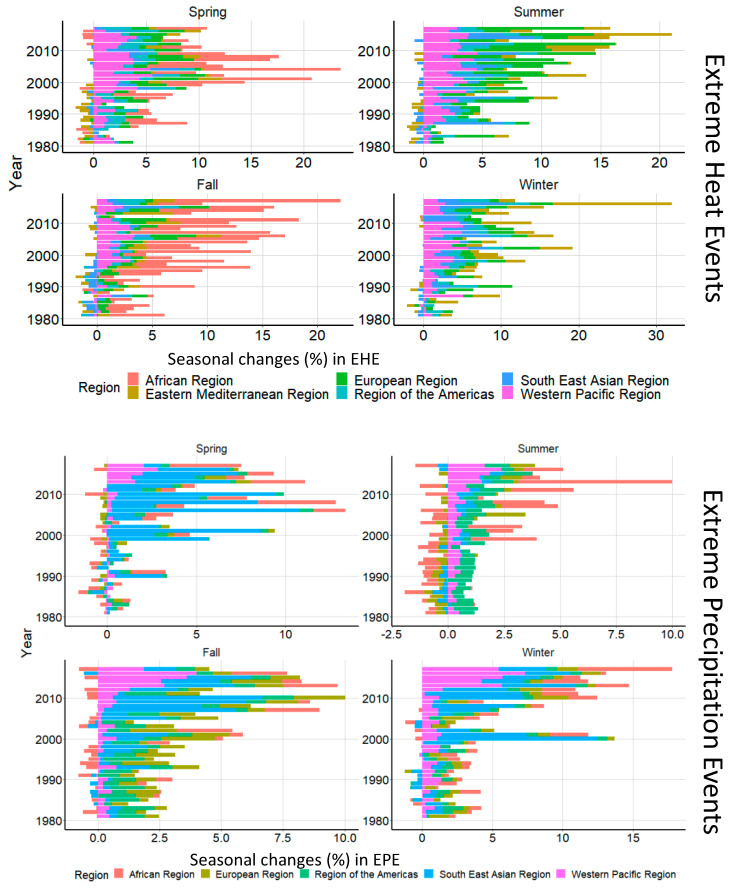
Seasonal changes in EHE (**top**) and EPE (**bottom**) during 1980–2017.

As depicted in Figure 3 and Supplementary Table S2, metropolitan locations in the African Region had the highest EHE frequency during the 2000s (median seasonal EHE (IQR): 10.5 (6.3–19.7), 9.3 (3.3–16.2), 12.3 (4.3–15.0), and 10.8 (6.0–16.6) for spring, summer, fall, and winter, respectively), while the Eastern Mediterranean Region had the highest EHE frequency during the 2010s (median seasonal EHE (IQR): 7.2 (5.1–12.4), 11.8 (8.1–18.9), (15.5 (10.6–20.4), and 4.9 (3.1–9.9), for spring, summer, fall, and winter, respectively). Likewise, metropolitan locations in the Western Pacific Region had the most pronounced increases in EPE during the 2010s (median seasonal EPE (IQR) (9.1 (5.0–18.7), 7.8 (4.0–13.9), 6.8 (4.0–10.2), 8.0 (5.0–16.5), for spring, summer, fall, and winter, respectively (Figure 2, Supplementary Table S2). When we repeated this analysis using ECMWF ERA5 reanalysis products, findings for EHE did not change, while the trends for EPE were no longer pronounced ( Supplementary Figure S2b). 

**Figure 4:**
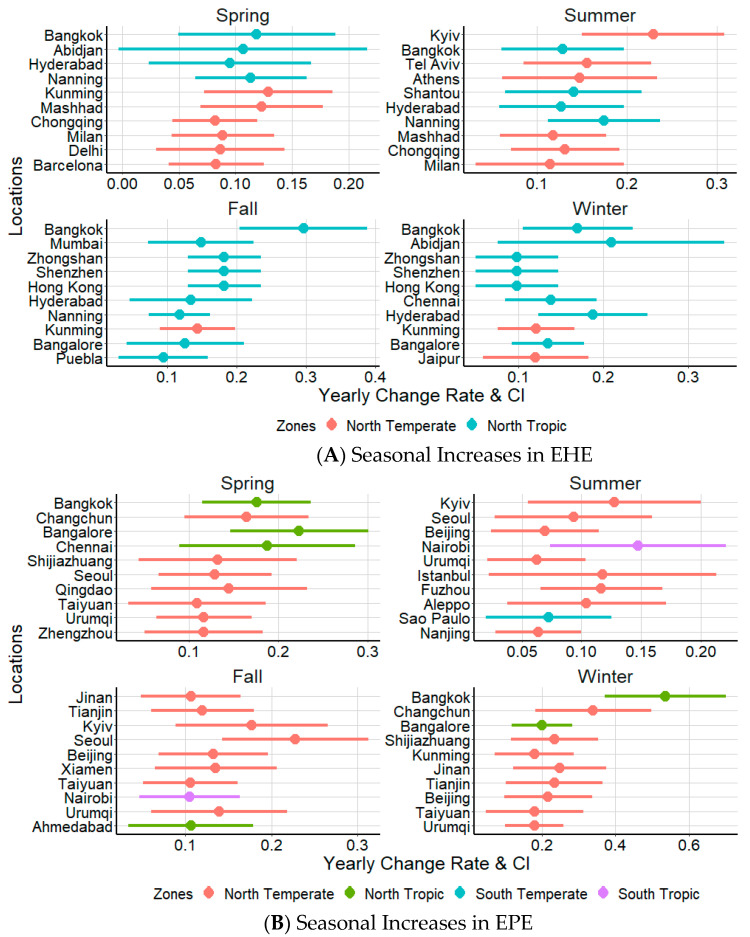
Ten metropolitan locations with the highest rate of seasonal increases in EHE (**top**) and EPE (**bottom**).

Based on regression analysis, we identified the top 10 metropolitan locations with the highest seasonal rate of increases in EHE and EPE frequencies (Figure 4). Six out of the top ten cities with the highest seasonal increases in EHE frequency during spring and summer are from North Temperate zones, while eight out of the top ten cities with the highest rate of increasing EHE frequency in fall and winter are from North Tropic zones (Figure 4A). In contrast, the majority of cities with the highest rate of increases in EPE were from North Temperate zones, irrespective of the season (Figure 4B).

When we considered cities individually, Bangkok had the fastest rate of seasonal increases in EHE during fall (0.297 EHE/Fall; 95%CI: 0.204–0.389), while Kyiv had the fastest rate of EHE increases during summer (0.229, 95%CI: 0.150–0.308). Likewise, Abidjan had the fastest rate of EHE increases during winter (0.209, 95%CI: 0.071–0.343), while the rates of increase were highest in Kunming during spring (0.119, 95%CI: 0.069–0.177). Overall, we observed higher rates of EHE increases during the summer and fall seasons. Cities such as Hyderabad and Bangkok experienced EHE increases across all seasons while the rate of EHE increases for other locations were confined to only one or two seasons (Figure 4A). For EPE, the highest wintertime increase was observed in Bangkok (0.535 EHEs/winter, 95%CI: 0.371–0.699), and Seoul during fall (0.228, 95%CI: 0.143–0.313). Likewise, the rate of EPE increases was highest in Bangalore during spring (0.224, 95%CI: 0.146–0.301) and in Nairobi during summer (0.148, 95%CI: 0.074–0.221). The rate of EPE increases were more pronounced during winter, with cities such as Urumqi experiencing increases during all seasons while cities such as Taiyuan, Seoul and Beijing experiencing increases during three out of four seasons (Figure 4B).

**Figure 5:**
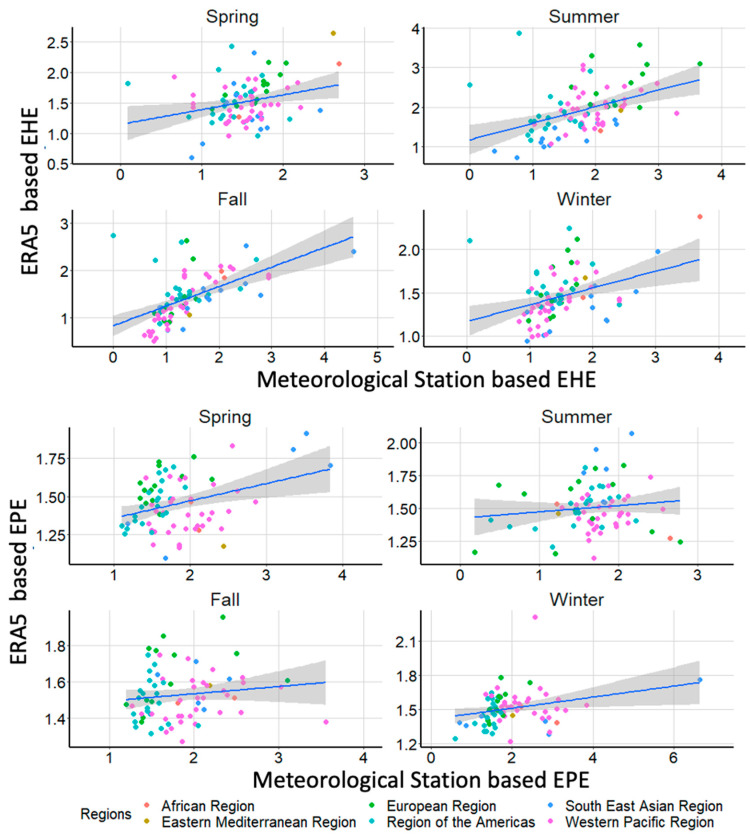
Correlation between weather station vs. ERA5 based EHE (**A**) top and EPE (**B**) bottom frequencies during 1980–2017.

We also explored the relationship between EHE/EPE calculated using weather station data vs. ERA5 reanalysis products (Figure 5). Overall, the agreement in EHE frequency between monitoring stations and ERA5 was highest during fall (r = 0.55) and lowest during spring (r = 0.28) (Figure 5A). Overall, the ERA5 based estimates tend to underestimate the actual frequency of EHE. Similarly, correlation of EPE frequency derived using monitoring station data and ERA5 reanalysis products were highest during spring (r = 0.38) and lowest during summer (r = 0.12) (Figure 5B). Overall, the agreement between monitoring stations and ERA5 based estimates were higher for EHE and lower for EPE.

## 4. Discussion

While the list of adverse human health impacts that are tied to the ongoing climate change continues to grow, the geographic distribution of populations that are exposed to extreme events and therefore, at risk of experiencing adverse health outcomes are lacking at a global scale. Taking into consideration both the rapid rise in urban populations and the disproportionate increases in EHE and EPE in major cities [30,31], we show that the number of individuals exposed to extreme events has increased by 500% in these global population centers since the 1980s. Our findings are of particular concern given that the most recent United Nations projections suggest that urban populations will continue to grow over the next decades, with the current share of urban populations increasing from 55% to 68% by 2050 [35]. Importantly, close to 90% of the projected 2.5 billion increase in urban populations will take place in Asia and Africa [55]. Our findings suggest that there is a pressing need to enhance adaptation strategies in these areas to deal with increases in populations exposed to extreme heat and precipitation events.

Our data showing increases in EPE across cities in the Western Pacific Region during the 2010s is consistent with Huang et al. [56], who tied special circulation patterns favorable to wintertime extreme precipitation events over South China in the 2010s. The large number of cities from the European Region and Western Pacific Region with noted increases in summertime EHE is consistent with recent studies focusing on these regions [57,58], as well as the noted increases in European summer heatwaves [59].

When we compared EHE results that were derived using weather station data to those derived using ERA5 reanalysis products, the agreement was considerably better for EHE compared to EPE. This is expected since simulating precipitation is generally much more challenging for models than simulating temperature [60]. Although the weather station-based calculation of exposed populations was based on 84 cities while the ERA5 reanalysis-based calculation included all 150 cities, we observed remarkable consistencies in relative increases in populations exposed to EHE between 1980 and 2017 (~500%). However, there was a significant difference in populations exposed to EPE when comparing the station-based measures as opposed to the ERA5 based measures (500% vs. 150%). Although, the ERA5 based estimates included 66 additional cities that did not have weather station-based estimates, it is unlikely that they all experienced reduced frequency of EPE. This suggest that ERA5 products underestimate precipitation considerably in these metropolitan locations. Focusing on specific regions, there was a better agreement between the reanalysis product and weather station based EHE for the European region, likely due to good spatial and temporal coverage of the station-based data. The Region of the Americas included both North and South America, which likely resulted in the lower agreement, although North America has a good spatial and temporal coverage of weather stations. Due to the relatively small number of stations over the African and Eastern Mediterranean, it was difficult to draw a meaningful conclusion comparing the two different approaches. Moreover, the WHO classification of the world region is not based on climatological regions, which further complicates the interpretation. These findings highlight the caveats of using exposure metrics that are derived solely based on modeled data in epidemiological studies.

There are several strengths of our study. This study focused on potential exposures to extreme heat and precipitation events in the 150 major cities of the world over the past four decades. We further analyzed how these exposures varied across WHO regions and latitudes, which provided valuable information for assessing regional disparities in exposure and potential health impacts. Our findings will inform public health preparedness, particularly across low- and middle-income countries where such data have been lacking. Our study has some limitations as well. First, only 84 out of the 150 cities had weather station level data that matched our selection criteria, which limited our analysis. To overcome this, we used ERA5 reanalysis products. However, limited agreement between ERA5 reanalysis products and weather station level extreme event data, particularly with regards to EPE, raises concerns about just relying on the ERA5 based analysis alone. Nevertheless, these findings also underscore the need for sustained monitoring stations to capture the expected continuation of increases in weather extremes in a warming world.

Given the linkage between exposure to extreme events and impaired health [1,2,3], our data showing rapid increases in urban population exposed to EHE and EPE underscores the need for community specific adaptation strategies. Since vulnerabilities to extreme weather events depend upon range of characteristics, such as sociodemographic factors including race, ethnicity, income, pre-existing health conditions, access to medical care, as well as capacity to adapt to these new set of hazards, municipalities across the globe need to invest in early warning systems. Given that currently available weather-based warnings with a week to ten-day lead time does not provide enough time to mobilize public health responses, these new set of early warnings should strive for seasonal to sub-seasonal lead time and should take into considerations location specific geographic and sociodemographic drivers of vulnerability, as well as spatial heterogeneity in exposures.

## 5. Conclusions

The confluence of disproportionate increases in the frequency of extreme weather events in city centers, and rapid rises in urban populations has turned major cities across the globe into flashpoints for climate change driven disease burden with a 500% increase in populations exposed to extreme heat and precipitation events over the past four decades. The projected increases in the frequency of extreme heat events and the continued rises in urban populations combined with ever increasing disparities in income, food, and healthcare access in urban areas will only worsen public health outcomes in these city centers in coming decades. Cities across the globe need to prepare for such increases in exposed populations and begin evaluating the efficacy of their adaptation strategies.

## Data Availability

The data used for this study is publicly available for everyone.

## Supplementary Materials

The following are available online at https://www.mdpi.com/1660-4601/18/3/1293/s1, Table S1: Details of 150 most populated included in the study. Table S2: Summary of frequency of EHE and EPE by Season, Decade and WHO Regions. Figure S1. Change of EHE (top) and EPE (bottom) exposed population in the last four decades using based on data from ERA5 reanalysis. FigureS2: Metropolitan locations with fastest increasing trend in EHE (top) and EPE (bottom) based on ERA5 reanalysis data.
