# Supplementary material for: Global Population Exposed to Extreme Events in the 150 Most Populated Cities of the World: Implications for Public Health
Source: Int J Environ Res Public Health. 2021 Feb 1;18(3):1293. doi: 10.3390/ijerph18031293 (PMC7908124; doi:10.3390/ijerph18031293)
Supplement: Supplementary file 1 [file ijerph-18-01293-s001.pdf]

Supplemental Table S1: Details of 150 most populated included in the study

Metropolitan Region	Country	Pop. (2017)	WHO Region	Zone
Tokyo	Japan	3.80E+07	Western Pacific Region	North Temperature
Delhi	India	2.57E+07	South East Asian Region	North Temperature
Shanghai	China	2.37E+07	Western Pacific Region	North Temperature
Sao Paulo	Brazil	2.11E+07	Region of the Americas	South Temperature
Mumbai	India	2.10E+07	South East Asian Region	North Tropic
Mexico City	Mexico	2.10E+07	Region of the Americas	North Tropic
Beijing	China	2.04E+07	Western Pacific Region	North Temperature
Osaka	Japan	2.02E+07	Western Pacific Region	North Temperature
Cairo	Egypt	1.88E+07	Eastern Mediterranean Region	North Temperature
New York City	United States	1.86E+07	Region of the Americas	North Temperature
Dhaka	Bangladesh	1.76E+07	South East Asian Region	North Temperature
Karachi	Pakistan	1.66E+07	Eastern Mediterranean Region	North Temperature
Buenos Aires	Argentina	1.52E+07	Region of the Americas	South Temperature
Kolkata	India	1.49E+07	South East Asian Region	North Tropic
Istanbul	Turkey	1.42E+07	European Region	North Temperature
Chongqing	China	1.33E+07	Western Pacific Region	North Temperature
Lagos	Nigeria	1.31E+07	African Region	North Tropic
Manila	Philippines	1.29E+07	Western Pacific Region	North Tropic
Rio de Janeiro	Brazil	1.29E+07	Region of the Americas	South Tropic
Guangzhou	China	1.25E+07	Western Pacific Region	North Tropic
Los Angeles	United States	1.23E+07	Region of the Americas	North Temperature
Moscow	Russia	1.22E+07	European Region	North Temperature
Kinshasa	Democratic Republic of the Congo	1.16E+07	African Region	South Tropic
Tianjin	China	1.12E+07	Western Pacific Region	North Temperature
Paris	France	1.08E+07	European Region	North Temperature
Shenzhen	China	1.07E+07	Western Pacific Region	North Tropic
Jakarta	Indonesia	1.03E+07	South East Asian Region	South Tropic
London	United Kingdom	1.03E+07	European Region	North Temperature
Bangalore	India	1.01E+07	South East Asian Region	North Tropic
Lima	Peru	9.90E+06	Region of the Americas	South Tropic
Chennai	India	9.89E+06	South East Asian Region	North Tropic
Seoul	South Korea	9.77E+06	Western Pacific Region	North Temperature
Bogotá	Colombia	9.76E+06	Region of the Americas	North Tropic
Nagoya	Japan	9.41E+06	Western Pacific Region	North Temperature
Johannesburg	South Africa	9.40E+06	African Region	South Temperature

Bangkok	Thailand	9.27E+06	South East Asian Region	North Tropic
Hyderabad	India	8.94E+06	South East Asian Region	North Tropic
Chicago	United States	8.74E+06	Region of the Americas	North Temperature
Lahore	Pakistan	8.74E+06	Eastern Mediterranean Region	North Temperature
Tehran	Iran	8.43E+06	Eastern Mediterranean Region	North Temperature
Wuhan	China	7.91E+06	Western Pacific Region	North Temperature
Chengdu	China	7.56E+06	Western Pacific Region	North Temperature
Dongguan	China	7.43E+06	Western Pacific Region	North Tropic
Nanjing	China	7.37E+06	Western Pacific Region	North Temperature
Ahmadabad	India	7.34E+06	South East Asian Region	North Tropic
Hong Kong	China	7.31E+06	Western Pacific Region	North Tropic
Ho Chi Minh City	Vietnam	7.30E+06	Western Pacific Region	North Tropic
Foshan	China	7.04E+06	Western Pacific Region	North Tropic
Kuala Lumpur	Malaysia	6.84E+06	Western Pacific Region	North Tropic
Baghdad	Iraq	6.64E+06	Eastern Mediterranean Region	North Temperature
Santiago	Chile	6.51E+06	Region of the Americas	South Temperature
Hangzhou	China	6.39E+06	Western Pacific Region	North Tropic
Riyadh	Saudi Arabia	6.37E+06	Eastern Mediterranean Region	North Temperature
Shenyang	China	6.32E+06	Western Pacific Region	North Temperature
Madrid	Spain	6.20E+06	European Region	North Temperature
Xi'an	China	6.04E+06	Western Pacific Region	North Temperature
Toronto	Canada	5.99E+06	Region of the Americas	North Temperature
Miami	United States	5.82E+06	Region of the Americas	North Temperature
Pune	India	5.73E+06	South East Asian Region	North Tropic
Belo Horizonte	Brazil	5.72E+06	Region of the Americas	South Tropic
Dallas	United States	5.70E+06	Region of the Americas	North Temperature
Surat	India	5.65E+06	South East Asian Region	North Tropic
Houston	United States	5.64E+06	Region of the Americas	North Temperature
Singapore	Singapore	5.62E+06	Western Pacific Region	North Tropic
Philadelphia	United States	5.59E+06	Region of the Americas	North Temperature
Kitakyushu	Japan	5.51E+06	Western Pacific Region	North Temperature
Luanda	Angola	5.51E+06	African Region	South Tropic
Suzhou	China	5.47E+06	Western Pacific Region	North Temperature
Harbin	China	5.46E+06	Western Pacific Region	North Temperature
Barcelona	Spain	5.26E+06	European Region	North Temperature
Atlanta	United States	5.14E+06	Region of the Americas	North Temperature
Khartoum	Sudan	5.13E+06	Eastern Mediterranean Region	North Tropic
Dar es Salaam	Tanzania	5.12E+06	African Region	South Tropic
Saint Petersburg	Russia	4.99E+06	European Region	North Temperature
Washington, D.C.	United States	4.96E+06	Region of the Americas	North Temperature

Abidjan	Cote d'Ivoire	4.86E+06	African Region	North Tropic
Guadalajara	Mexico	4.84E+06	Region of the Americas	North Tropic
Yangon	Myanmar	4.80E+06	South East Asian Region	North Tropic
Alexandria	Egypt	4.78E+06	Eastern Mediterranean Region	North Temperature
Ankara	Turkey	4.75E+06	European Region	North Temperature
Kabul	Afghanistan	4.63E+06	Eastern Mediterranean Region	North Temperature
Qingdao	China	4.57E+06	Western Pacific Region	North Temperature
Chittagong	Bangladesh	4.54E+06	South East Asian Region	North Tropic
Monterrey	Mexico	4.51E+06	Region of the Americas	North Temperature
Sydney	Australia	4.51E+06	Western Pacific Region	South Temperature
Dalian	China	4.49E+06	Western Pacific Region	North Temperature
Xiamen	China	4.43E+06	Western Pacific Region	North Temperature
Zhengzhou	China	4.39E+06	Western Pacific Region	North Temperature
Boston	United States	4.25E+06	Region of the Americas	North Temperature
Melbourne	Australia	4.20E+06	Western Pacific Region	South Temperature
Brasília	Brazil	4.16E+06	Region of the Americas	South Tropic
Jiddah	Saudi Arabia	4.08E+06	Eastern Mediterranean Region	North Tropic
Phoenix	United States	4.06E+06	Region of the Americas	North Temperature
Ji'nan	China	4.03E+06	Western Pacific Region	North Temperature
Montréal	Canada	3.98E+06	Region of the Americas	North Temperature
Shantou	China	3.95E+06	Western Pacific Region	North Tropic
Nairobi	Kenya	3.91E+06	African Region	South Tropic
Medellín	Colombia	3.91E+06	Region of the Americas	North Tropic
Fortaleza	Brazil	3.88E+06	Region of the Americas	South Tropic
Kunming	China	3.78E+06	Western Pacific Region	North Temperature
Changchun	China	3.76E+06	Western Pacific Region	North Temperature
Changsha	China	3.76E+06	Western Pacific Region	North Temperature
Recife	Brazil	3.74E+06	Region of the Americas	South Tropic
Rome	Italy	3.72E+06	European Region	North Temperature
Zhongshan	China	3.69E+06	Western Pacific Region	North Tropic
Cape Town	South Africa	3.66E+06	African Region	South Temperature
Detroit	United States	3.64E+06	Region of the Americas	North Temperature
Hanoi	Vietnam	3.63E+06	Western Pacific Region	North Temperature
Tel Aviv	Israel	3.61E+06	European Region	North Temperature
Porto Alegre	Brazil	3.60E+06	Region of the Americas	South Temperature
Kano	Nigeria	3.59E+06	African Region	North Tropic
Salvador	Brazil	3.58E+06	Region of the Americas	South Tropic
Faisalabad	Pakistan	3.57E+06	Eastern Mediterranean Region	North Temperature
Berlin	Germany	3.56E+06	European Region	North Temperature
Aleppo	Syria	3.56E+06	Eastern Mediterranean Region	North Temperature
Dakar	Senegal	3.52E+06	African Region	North Tropic
Casablanca	Morroco	3.51E+06	Eastern Mediterranean Region	North Temperature
Urumqi	China	3.50E+06	Western Pacific Region	North Temperature

Taiyuan	China	3.48E+06	Western Pacific Region	North Temperature
Curitiba	Brazil	3.47E+06	Region of the Americas	South Temperature
Jaipur	India	3.46E+06	South East Asian Region	North Temperature
Shizuoka	Japan	3.37E+06	Western Pacific Region	North Temperature
Hefei	China	3.35E+06	Western Pacific Region	North Temperature
San Francisco	United States	3.30E+06	Region of the Americas	North Temperature
Fuzhou	China	3.28E+06	Western Pacific Region	North Temperature
Shijiazhuang	China	3.26E+06	Western Pacific Region	North Temperature
Seattle	United States	3.25E+06	Region of the Americas	North Temperature
Addis Ababa	Ethiopia	3.24E+06	African Region	North Tropic
Nanning	China	3.23E+06	Western Pacific Region	North Tropic
Lucknow	India	3.22E+06	South East Asian Region	North Temperature
Busan	South Korea	3.22E+06	Western Pacific Region	North Temperature
Wenzhou	China	3.21E+06	Western Pacific Region	North Temperature
Ibadan	Nigeria	3.16E+06	African Region	North Tropic
Ningbo	China	3.13E+06	Western Pacific Region	North Temperature
San Diego	United States	3.11E+06	Region of the Americas	North Temperature
Milan	Italy	3.10E+06	European Region	North Temperature
Yaounde	Cameroon	3.07E+06	African Region	North Tropic
Athens	Greece	3.05E+06	European Region	North Temperature
Wuxi	China	3.05E+06	Western Pacific Region	North Temperature
Campinas	Brazil	3.05E+06	Region of the Americas	South Temperature
Izmir	Turkey	3.04E+06	European Region	North Temperature
Kanpur	India	3.02E+06	South East Asian Region	North Temperature
Mashhad	Iran	3.01E+06	Eastern Mediterranean Region	North Temperature
Puebla	Mexico	2.98E+06	Region of the Americas	North Tropic
Sana'a	Yemen	2.96E+06	Eastern Mediterranean Region	North Tropic
Santo Domingo	Domican Republic	2.95E+06	Region of the Americas	North Tropic
Douala	Cameroon	2.94E+06	African Region	North Tropic
Kiev	Ukraine	2.94E+06	European Region	North Temperature
Guatemala City	Guatemala	2.92E+06	Region of the Americas	North Tropic
Caracas	Venezuela	2.92E+06	Region of the Americas	North Tropic

Supplemental Table S2: Summary of frequency of EHE and EPE by Season, Decade and WHO Regions

Region	Decade	EHE (IQR) Winter		Spring		Summer		Fall	
Region of the Americas	1980s	2.0	(1.0, 5.0)	3.0	(1.0,5.0)	2.0	(0.3,5)	2.0	(1.0,4.0)
	1990s	3.0	(1.0,5.0)	2.6	(1.0,5.0)	2.5	(0,6.0)	2.1	(1.0,5.0)
	2000s	3.0	(1.0,6.0)	4.0	(1.1,6.0)	2.0	(0,5.0)	2.0	(1.0,5.0)
	2010s	4.0	(2.0,7.0)	4.0	(2.0,7.3)	3.9	(0,7.0)	4.0	(2.0,8.0)
European Region	1980s	3.0	(1.0,5.0)	2.0	(0,4.0)	2.0	(1.0,5.0)	2.0	(1.0,4.0)
	1990s	3.9	(2.0,6.0)	4.0	(2.0,7.0)	5.0	(1.0,8.0)	3.0	(1.0,4.0)
	2000s	4.1	(2.0,7.0)	5.2	(3.0,8.0)	**7.0**	(4.0,14.0)	3.0	(1.0,4.0)
	2010s	4.0	(1.5,8.0)	**7.1**	(3.0,12.0)	**9.1**	(4.0,17.0)	4.0	(1.0,8.0)
Western Pacific Region	1980s	2.0	(1.0,4.0)	2.1	(1.0,5.0)	2.0	(1.0,5.0)	1.1	(0,3.0)
	1990s	4.0	(2.0,5.7)	3.0	(1.2,5.0)	4.0	(2.0,7.0)	3.0	(1.0,5.0)
	2000s	5.0	(2.0,8.0)	6.0	(3.1,9.5)	7.0	(3.0,12.0)	4.1	(2.0,7.0)
	2010s	3.0	(1.0,6.1)	4.4	(2.0,8.0)	6.0	(2.0,12.0)	2.4	(0,8.0)
South East Asian Region	1980s	1.1	(0,3.0)	2.3	(0.8,5.5)	1.1	(0,3.0)	2.4	(0.8,6.2)
	1990s	2.3	(0.8,5.7)	2.6	(1.0,5.6)	2.2	(0,5.1)	1.1	(0,3.2)
	2000s	**7.0**	(3.0,11.4)	4.0	(1.3,7.5)	2.4	(0,6.8)	4.6	(2.1,11.8)
	2010s	6.1	(2.1,14.3)	4.4	(0,9.7)	3.1	(0,11.0)	**6.0**	(0,15.5)
African Region	1980s	2.4	(0.9,4.5)	2.1	(0,3.6)	1.2	(0,3.2)	1.5	(0,2.9)
	1990s	4.9	(2.3,6.7)	4.2	(1.5,7.3)	2.8	(0,9.4)	3.4	(1.2,5.5)
	2000s	**10.5**	(6.3,19.7)	**9.3**	(3.3,16.2)	**12.3**	(4.3,15.0)	**10.8**	(6.0,16.6)
	2010s	5.8	(2.7,7.4)	0.6	(0,8.0)	4.6	(0,12.3)	3.2	(0,6.2)
Eastern Mediterranean	1980s	2.0	(1.3,4.8)	2.0	(1.0,5.1)	3.0	(1.3,4.8)	3.0	(2.0,3.8)
	1990s	3.6	(2.3,5.8)	3.5	(2.0,5.1)	4.6	(2.0,9.3)	4.0	(2.3,4.8)
	2000s	**6.0**	(3.3,8.0)	**10.5**	(9.3,16.7)	5.0	(3.3,7.3)	3.5	(1.3,5.5)
	2010s	**7.2**	(5.1,12.4)	**11.8**	(8.1,18.9)	**15.5**	(10.6,20.4)	4.9	(3.1,9.9)

Region	Decade	EPE (IQR) Winter		Spring		Summer		Fall	
Region of the America	1980s	4.0	(2.0,5.0)	4.0	(2.0,6.0)	4.0	(2.0,5.0)	4.0	(3.0,6.0)
	1990s	4.8	(3.0,6.0)	4.0	(3.0,6.0)	4.0	(2.0,5.0)	4.0	(2.0,6.0)

	2000s	4.0	(2.0,6.0)	4.0	(3.0,6.0)	4.0	(2.0,6.0)	4.0	(2.2,6.0)
	2010s	5.0	(3.0,6.0)	4.9	(3.0,7.0)	5.0	(2.3,7.0)	4.0	(2.8,5.0)
European Region	1980s	4.0	(3.0,6.0)	4.0	(3.0,6.0)	4.0	(1.7,6.0)	4.0	(2.0,5.0)
	1990s	4.0	(2.0,6.0)	4.0	(3.0,5.7)	4.0	(2.0,5.0)	4.0	(2.8,6.6)
	2000s	5.0	(3.0,7.0)	4.0	(2.0,6.0)	3.1	(2.0,6.0)	5.0	(3.0,7.0)
	2010s	4.8	(3.0,7.4)	4.9	(2.0,7.8)	4.6	(2.0,8.0)	5.0	(3.0,7.0)
Western Pacific Region	1980s	3.0	(2.0,5.0)	4.0	(3.0,6.0)	4.0	(3.0,6.0)	4.0	(3.0,6.0)
	1990s	4.0	(2.0,6.3)	4.0	(3.0,6.0)	5.0	(3.0,6.0)	4.0	(3.0,6.0)
	2000s	5.0	(3.1,7.0)	4.0	(3.0,6.0)	5.0	(3.0,6.0)	4.5	(3.0,6.0)
	2010s	**9.1**	(5.0,18.7)	**7.8**	(4.0,13.9)	**6.6**	(4.0,10.2)	**8.0**	(5.0,16.5)
South East Asian Region	1980s	1.1	(0,3.1)	2.1	(0.8,3.4)	4.2	(3.0,5.3)	3.4	(1.8,5.4)
	1990s	2.0	(1.0,3.8)	3.1	(0,5.2)	5.0	(2.1,6.8)	4.2	(3.0,5.6)
	2000s	3.7	(0,18.9)	**8.5**	(1.1,18.0)	6.2	(3.4,8.3)	6.2	(2.3,11.5)
	2010s	6.0	(0,24.1)	**14.6**	(0,21.8)	5.6	(1.4,9.5)	**7.7**	(2.3,13.3)
African Region	1980s	3.0	(2.0,6.3)	3.5	(2.8,5.3)	4.5	(2.0,7.0)	5.5	(3.8,7.3)
	1990s	5.0	(3.8,6.0)	2.5	(1.2,6.0)	4.0	(3.0,5.0)	3.0	(2.0,5.5)
	2000s	3.0	(1.6,5.6)	**9.3**	(5.9,12.2)	6.8	(2.8,16.2)	**7.8**	(3.7,9.1)
	2010s	**7.1**	(3.4,16.8)	**8.2**	(0,11.3)	**8.4**	(1.3,17.2)	5.6	(0,11.3)
Eastern Mediterr anean	1980s	2.5	(0.4,5.1)	3.3	(0.4,4.7)	0	(0,0)	2.0	(1.2,4.4)
	1990s	3.5	(2.7,5.2)	4.8	(2.5,6.9)	0	(0,1.1)	3.9	(2.3,5.6)
	2000s	5.3	(4.0,13.9)	3.1	(2.3,5.4)	3.1	(1.3,4.1)	4.1	(3.2,7.9)
	2010s	4.2	(1.3,6.3)	**7.7**	(4.0,10.4)	6.7	(2.6,13.4)	3.7	(1.7,14.7)

Supplemental Equations:

$$YAEP_N = \frac{\sum_{i=1}^{n} EP_{iN}}{n} = \frac{\sum_{i=1}^{n} (E_{iN} \bullet P_{iN})}{n} \quad \ldots\ldots\ldots\ldots\ldots\ldots\ldots\ldots\ldots\ldots \quad (3)$$

Where $YAEP_N$ = the yearly average of exposed population; E_{iN} = the EHE in city i and year N ; P_{iN} = population in city i and year N, and n= total number of cities included in the analysis.

Supplemental Figure S1. Change of EHE (top) and EPE (bottom)exposed population in the last four decades using based on data from ERA5 reanalysis.

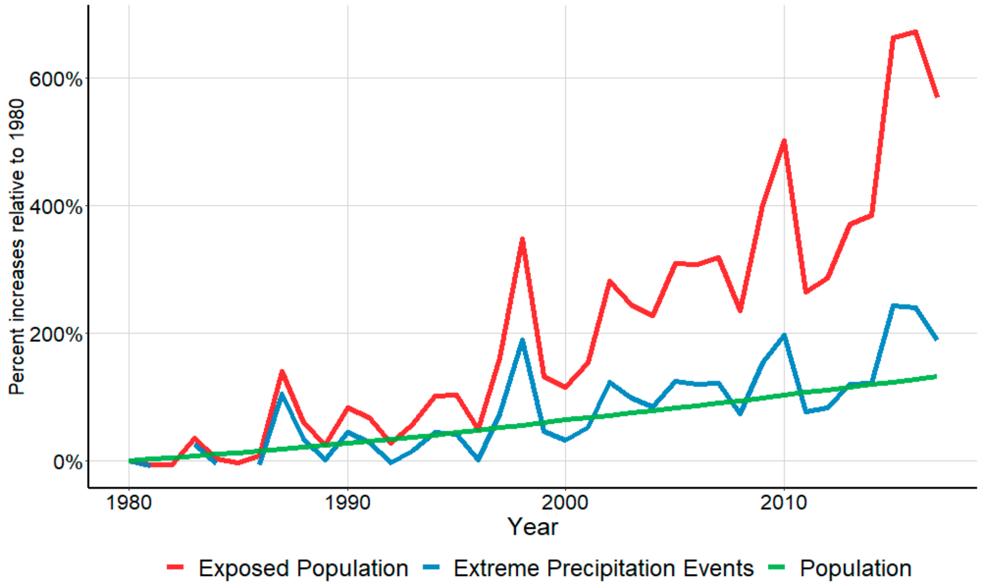

(a)

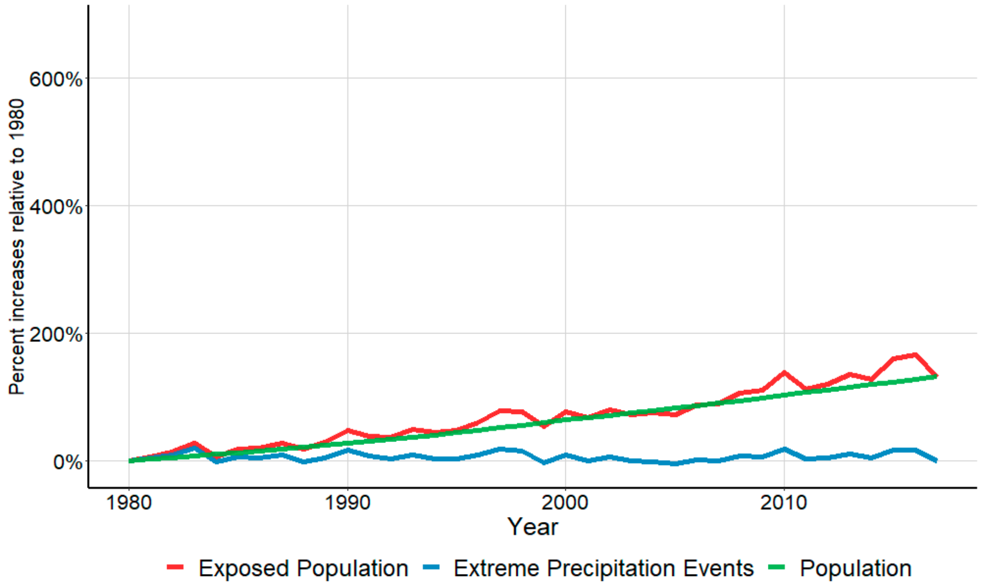

(b)

Supplemental FigureS2 : Metropolitan locations with fastest increasing trend in EHE (top) and EPE (bottom) based on ERA5 reanalysis data

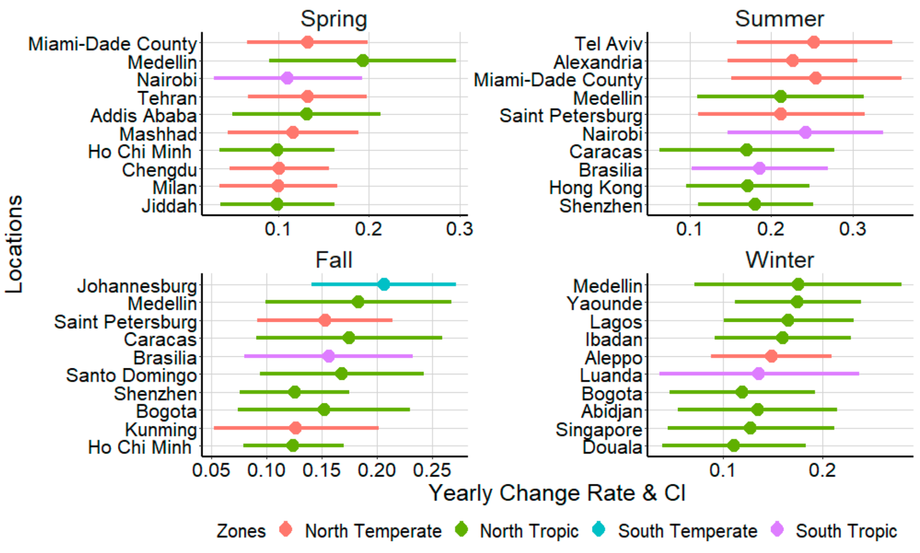

(a)

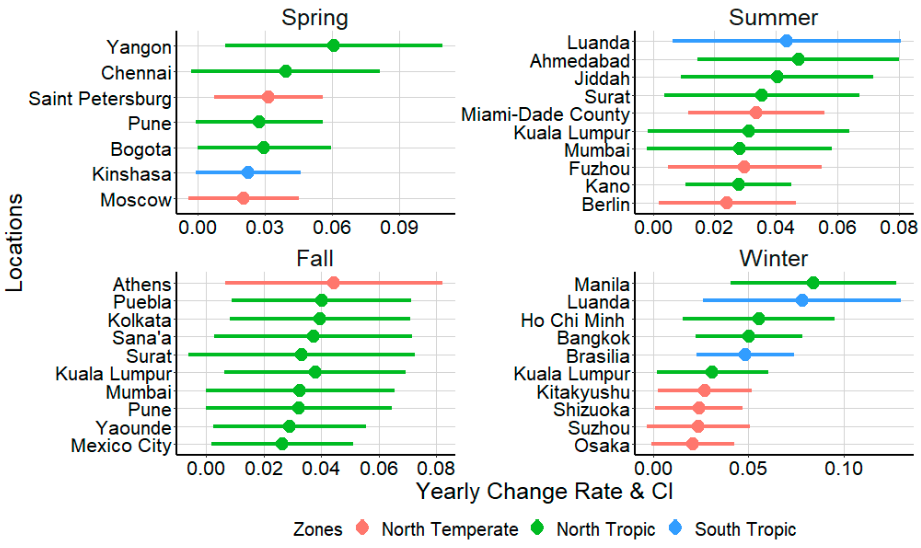

(b)
